# Sisyphus observed: Unraveling the high ATP usage of an RNA chaperone

**DOI:** 10.1016/j.jbc.2021.100265

**Published:** 2021-02-11

**Authors:** Elizabeth C. Duran, Nils G. Walter

**Affiliations:** Department of Chemistry and Center for RNA Biomedicine, University of Michigan, Ann Arbor, Michigan, USA

**Keywords:** ATP hydrolysis, group I intron ribozyme, RNA chaperone, RNA folding

## Abstract

DEAD-box proteins are nonprocessive RNA helicases that can function as RNA chaperones by coupling ATP binding and hydrolysis to structural reorganization of RNA. Here, Jarmoskaite *et al.* quantify the ATP utilization of an RNA chaperone during refolding of a misfolded ribozyme substrate. Strikingly, 100 ATP hydrolysis events are needed per successfully refolded ribozyme, suggesting that each round of unfolding requires ten ATP molecules, since 90% of substrate unfolding cycles only lead back to the kinetically favored misfolded state. This near-Sisyphean effort reveals a potentially conserved model for RNA reorganization by RNA chaperones.

The three-dimensional structure of a folded RNA is crucial to its processing, localization, and function in diverse cellular environments. As a polymer capable of forming numerous hydrogen bonds, stacking, and ionic interactions, the folding pathways of RNA are far from linear. Even more than proteins, with only four building blocks, RNAs can sample large numbers of thermodynamically exceedingly stable, yet often nonfunctional states that may have limited biological utility. As a countermeasure, energy-dependent molecular chaperones have evolved throughout biology to remodel their molecular substrates into functional conformations. These molecular chaperones couple ATP hydrolysis to physical interactions with their misfolded targets that facilitate the desired structural reorganization into the functional state. Despite their prominent role in cellular homeostasis, details remain largely unknown about how much fuel is required to power productive substrate rearrangement and what the nature of those rearrangements is, *e.g.*, either ordered or stochastic. Answers have been difficult to ascertain because of the substrate promiscuity and transient interactions characteristic of RNA helicases. Addressing these questions promises to provide insights into the many crucial metabolic processes such as splicing, RNA quality control pathways, and translation regulation mechanisms that rely on RNA helicase-driven remodeling.

The largest class of RNA helicases are DEAD-box proteins, named after a conserved amino acid sequence D-E-A-D. In general, these proteins couple the energy from ATP binding and hydrolysis to conformational changes that lead to nonprocessive unwinding and translocation of double-stranded RNA (1, 2). Some DEAD-box proteins additionally couple these transient unwinding and translocation activities to promote RNA substrate unfolding and refolding. One example of this is the DEAD-box protein CYT-19, which remodels group I and group II introns to promote splicing (3, 4). This activity has been well characterized in a group I intron ribozyme from *Tetrahymena thermophila* (5, 6). In over a decade of careful investigation of the CYT-19 chaperone activities, the Russell group has characterized its ATP-dependent interactions with both the native and misfolded ribozyme and determined which long-range contact disruptions within the misfolded state lead to global unfolding (4, 6, 7, 8). In short, CYT-19 accelerates ribozyme refolding into the native, catalytically active state by disrupting the kinetically trapped misfolded state (Fig. 1). Yet despite extensive study, mechanistic details about this CYT-19-driven process such as the number of ATP-dependent steps required as well as the number of local disruptions that lead to productive ribozyme remodeling have remained unclear.

**Figure 1:**
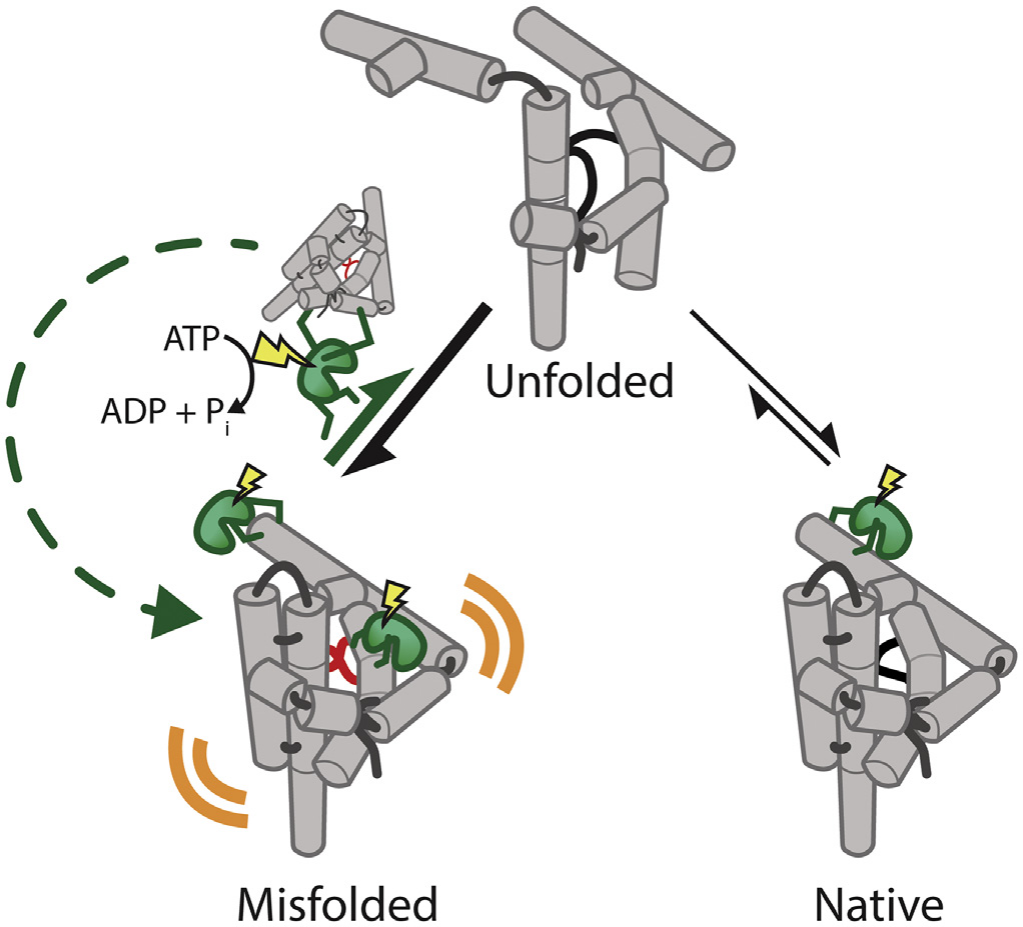
**CYT-19 ATP utilization model during *Tetrahymena* ribozyme unfolding.** CYT-19 (*green*) couples energy from ATP binding and hydrolysis (*yellow*) to bind exposed helices of both the native and misfolded ribozyme (*grey*). Thermal disruptions (*orange*) of local structural elements in the more open, misfolded state (featuring red crossed strands) make it more accessible to CYT-19. Continuous rounds of ATP-dependent unwinding by CYT-19 lead to global unfolding of the ribozyme such that it can eventually re-enter the folding pathway toward the kinetically less favored native state.

In their new work, Jarmoskaite *et al.* (9) implemented a set of experiments to quantify the ATP utilization of CYT-19 during ribozyme remodeling. Experiments using the cleavage of a short radiolabeled oligonucleotide by the functionally folded ribozyme report on the population of refolded ribozyme for a range of protein and ATP concentrations tested. By measuring the CYT-19 ATPase rate during ribozyme refolding, the authors determined the number of ATP molecules hydrolyzed per ribozyme molecule refolded. Implementing this strategy, the authors find that ∼300 ATP molecules are hydrolyzed for each ribozyme successfully refolding. This is a striking amount of energy consumed, since global unfolding of that misfolded state is estimated to only require about ten ATPs per ribozyme. Why is this enzyme so inefficient?

To answer this question, the authors further investigated what portion of the ATP hydrolysis activity is required for productive ribozyme remodeling. By controlling for background ATP hydrolysis that does not contribute to remodeling, they estimate that ∼100 molecules of ATP are hydrolyzed during productive refolding of a single misfolded ribozyme. This suggests that the majority of the ATP hydrolysis activity is spent on futile interactions with the ribozyme that do not lead to structural reorganization. Further investigation of the ATP consumption rate reveals that, while it does not vary much with either protein or ATP concentration, it does depend on ribozyme structure stability. The authors find that increasing the stability of the misfolded ribozyme leads to an increase in ATP utilization and, conversely, adding destabilizing mutations leads to a decrease. This complements previous findings showing that CYT-19’s ATPase activity is activated by less compact, and presumably more accessible, ribozyme tertiary structures (4). Together their findings implicate a remodeling mechanism by this DEAD-box protein in which ATP hydrolysis is powering stochastic scanning of both the folded and misfolded ribozyme states for thermodynamic instability (Fig. 1). Once the protein finds an exposed loop on the misfolded ribozyme with which to interact, the ATP-dependent steps powering substrate unfolding are repeated as many times as needed to unfold the misfolded intermediate sufficiently until it shuttles into a new folding path. Since previous work has suggested that only one in ten refolding cycles leads toward the properly folded, functionally active state of the *T. thermophila* ribozyme, much ATP ends up being consumed (Fig. 1). In this model, the CYT-19 chaperone acts as a tertiary structure surveillance system that targets spontaneous disruptions to stable tertiary structures of substrate clients.

The emerging mechanism of CYT-19 substrate unfolding is reminiscent of the Greek myth of Sisyphus, in which the deceitful king is eternally condemned by Zeus to the futile task of rolling a boulder up a hill only to have it fall just short of reaching the top. Fortunately, unlike Sisyphus, CYT-19 finally succeeds in resolving the misfolded ribozyme after an average of approximately nine futile cycles (Fig. 1). Whether this excessive ATP consumption model is recapitulated *in vivo* remains to be determined as it is unclear how the ribozyme’s native and misfolded states partition in the cellular environment. Future experiments will need to resolve the *in vivo* population of the misfolded state and how that is coupled to transcription rate and concentration of CYT19 as well as other RNA chaperones that may be able to resolve it into the native state.

It would be reasonable to assume that other DEAD-box and similar DEAH-box proteins with RNA chaperone activities also display ATP usage in steps that are both productive and nonproductive in RNA reorganization. Delineating ATP usage as done for CYT-19 in this work could help inform a unifying picture of how these molecular chaperones reorganize their targets, thereby mechanistically underpinning the multitude of RNA processing pathways in which they are needed. During RNA splicing, for example, insights like this would be highly valuable in understanding the RNA secondary structure rearrangements during both the early assembly and late catalytic stages, which are largely driven by RNA helicases with chaperone activities (10). Future work on RNA chaperones should aim to dissect the RNA-linked ATP utilization activities for a variety of RNA targets and quantify the effects of accessory protein partners on those activities as a way to uncover the RNA metabolic pathways they modulate and perhaps unveil novel regulatory mechanisms under RNA chaperone control.

## Conflict of interest

The authors declare that they have no conflicts of interest with the contents of this article.
